# Association between High-Sensitive C-Reactive Protein and the Development of Liver Damage in Japanese Male Workers

**DOI:** 10.3390/ijerph18062985

**Published:** 2021-03-14

**Authors:** Reiko Kuroda, Kazuhiro Nogawa, Yuuka Watanabe, Hideki Morimoto, Kouichi Sakata, Yasushi Suwazono

**Affiliations:** 1 Department of Occupational and Environmental Medicine, Graduate School of Medicine, Chiba University, Chiba 260-8670, Japan; reiko-luna@umin.ac.jp (R.K.); nogawa@chiba-u.jp (K.N.); watanabe155@chiba-u.jp (Y.W.); morimoto@oh-handling.com (H.M.); sakata_ko@khi.co.jp (K.S.); 2 Division for Environment, Health and Safety, The University of Tokyo, Tokyo 113-8654, Japan

**Keywords:** high-sensitive C-reactive protein, workload, liver damage, aminotransferase, cohort studies

## Abstract

Background: The aim of this study was to determine whether a causative relationship exists between the development of liver damage and increased high-sensitivity C-reactive protein (HsCRP) levels by long-term follow-up in Japanese workers. Methods: The target participants comprised 7830 male workers in a Japanese steel company. The prospective cohort study was performed over a 6-year period, and annual health screening information was analyzed by pooled logistic regression. The endpoint, regarded as the development of liver damage, was defined as aspartate aminotransferase (AST) ≥ 40 IU/L. Results: A significant relationship between the development of liver damage and increased HsCRP levels was observed after adjusting for confounding factors such as various physiological and blood chemistry parameters and lifestyle factors. The odds ratio of a 1.5-fold increase in HsCRP was 1.07 (95% confidence interval: 1.03–1.10, *p* < 0.001). Conclusions: The results suggested that an increase of HsCRP is associated with the development of liver damage.

## 1. Introduction

The liver plays a central role in the metabolism of glucose and lipids. Numerous recent studies have reported an association of liver damage with the development of metabolic syndrome (MetS), cardiovascular disease, and type 2 diabetes [1,2,3,4]. In particular, nonalcoholic fatty liver disease (NAFLD) is a manifestation of the hepatic consequence of metabolic syndrome associated with obesity, insulin resistance, and dyslipidemia, all of which are features of MetS [5,6]. On the other hand, recently epidemiological studies have suggested that low-grade inflammation is associated with the development of MetS, diabetes, and atherothrombosis [7,8,9,10,11]. High-sensitive C-reactive protein (HsCRP), a general marker of acute and systemic inflammation, is an independent predictor of future cardiovascular events, stroke, and MetS [7,8,12]. Many investigations have shown an association between HsCRP and hepatic steatosis [13,14,15,16], one of them demonstrated that higher levels of C-reactive protein (CRP) reflected the severity of hepatic fibrosis in cases of nonalcoholic steatohepatitis [15]. Moreover, many cross-sectional studies [14,17,18,19,20,21,22,23] have demonstrated that high levels of CRP are related to abnormal liver function tests, such as elevated aminotransferase, gamma-glutamyltransferase, and alkaline phosphatase.

Regarding the association between increased CRP values and the development of atherosclerotic disease such as cardiovascular disease and stroke, an earlier study postulated an effect of HsCRP on the development of liver damage [21]. A recent study suggested that the inflammatory microenvironment caused by steatosis leads to both amplification of hepatic steatosis and potentially increased synthesis and release of CRP by the liver [14,24]. However, little research has focused on the association between increased CRP levels and the development of liver damage.

To our knowledge, few cohort studies have focused on this issue. Although no study has documented a causal association between increased HsCRP levels and elevate aminotransferase, a recent study showed that even within the normal range, higher HsCRP levels increased the risk of developing NAFLD in healthy middle-aged men [25]. Since CRP and liver function indices are generally measured in routine clinical practice or medical checkups, proof of the existence of a causal association between increased levels of HsCRP and elevated aminotransferase might be useful for early detection of future liver damage, a possible risk factor of metabolic syndrome (MetS), cardiovascular disease, and type 2 diabetes. Therefore, it is important to establish the causative relationship between HsCRP and the development of liver damage.

Against this background, we performed this prospective cohort study to determine the relationship between increased HsCRP levels and the development of liver damage as manifested by increased serum aminotransferase level.

## 2. Materials and Methods

### 2.1. Study Population

**Figure 1:**
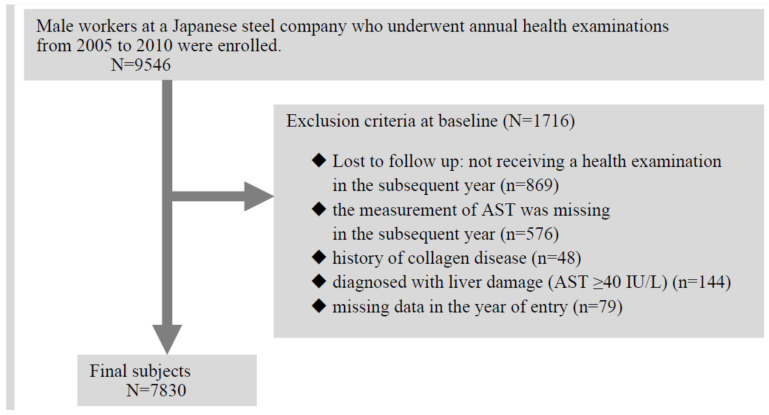
Flow diagram of study subjects.

This prospective cohort study included observations made over a 6-year period from 2005 to 2010. A total of 7830 subjects out of a possible 9546 male workers at a Japanese steel company were enrolled. Details of the study procedure have been described elsewhere [26]. The following individuals were excluded from this study: those who did not receive a health examination in the subsequent year (869 men), those for whom the measurement of aspartate aminotransferase (AST) was missing in the subsequent year (576 men), those who had a history of collagen disease (48 men), those who had been diagnosed with liver damage based on the criteria in the present study (144 men), and those with any missing data in the year of entry (79 men) (Figure 1). The study protocol was approved by the Ethics Review Board of the Graduate School of Medicine, Chiba University.

### 2.2. Measurements

All participants underwent health examinations between 09:00 and 15:00 throughout the whole study period; before the health examination, they fasted for at least 30 min and did not undertake any heavy physical activity. Occupational physicians performed health examinations and medical history interviews each year. A detailed description of the measurements has been published previously [26].

The endpoint in this study, liver damage, was defined as a value of AST higher or equal to 40 IU/L according to earlier studies [27,28].

The serum HsCRP level was measured by the latex turbidimetric immunoassay method [29]. The minimum detectable concentration and interassay coefficient of variation for HsCRP were 0.02 mg/dL and 2.43%, respectively.

We measured age in years, body mass index (BMI; weight in kg/(height in m) ^2), systolic blood pressure, diastolic blood pressure, HsCRP, high-density lipoprotein cholesterol (HDL-C), HbA1c, AST, creatinine, and uric acid. To take into account both systolic and diastolic blood pressures, we assessed blood pressure indexes based on mean arterial pressure. That was calculated using the formula: (systolic blood pressure + diastolic blood pressure × 2)/3 [30]. Self-administered questionnaires were used to obtain information on alcohol consumption, job schedule type, smoking status, habitual exercise, dietary habits, and job stress during the annual health examination. In the question about alcohol consumption, participants provided answers regarding (1) the number of alcohol drinking days per week (0–7) and (2) the quantity of their mean daily consumption based on the traditional Japanese sake (rice wine) unit of alcohol beverage, “gou”. One “gou” (180 mL) of sake, containing approximately 22 g of ethanol, is equivalent to 500 mL of beer, 60 mL of whiskey, 180 mL of wine, or 110 mL of shochu (distilled spirits). Weekly alcohol intake was calculated by multiplying the frequency of alcohol consumption by the quantity. The other variables as confounders include: (i) type of job schedule (as daytime, three-shift work, two-shift work, or other); (ii) smoking habit (non-smoker or smoker); (iii) habitual exercise (none, 1–2 times/month, 1–2 times/week, 3–4 times/week, or ≥ 5 times/week); (iv) preparation of meals (cooked by oneself, cooked by family member, eating out or catering, eating in a dormitory, or other); (v) soft-drink consumption (rare, occasional, or frequent); (vi) snacking between meals (rare, occasional, or frequent). Job-related stress was evaluated by the Brief Job Stress Questionnaire [31], including job demand, job control, interpersonal relationship, and compatibility. All covariates were updated whenever the annual health examination was performed.

### 2.3. Statistical Analysis

The HsCRP, HDL-C, HbA1c, AST, creatinine, and uric acid values were log-transformed to normalize their skewed distribution. The incidence rate of liver damage was calculated as the number of subjects who developed liver damage divided by the total number of person-years of observation (from 2005 to 2010). The relationship between increased HsCRP levels and the development of liver damage was evaluated with a pooled logistic regression analysis. Log HsCRP using a base of 1.5, age, alcohol consumption, BMI, mean blood pressure, log-transformed HDL-C, HbA1c, creatinine, and uric acid using a base of 1.5, job schedule type, smoking status, habitual exercise, dietary habits, and job stress were included simultaneously in the statistical model and selected by a forward stepwise method.

In a pooled regression analysis, each 1-year examination interval was treated as a mini follow-up study. The parameters obtained at the beginning of each 1-year follow-up were linked to the development of liver damage at the end of the follow-up. Then, these 1-year follow-ups were pooled, and the relationships between parameters obtained in the development of liver damage were analyzed using a logistic-regression model [32].

All analyses were performed using IBM SPSS 19J statistical software (IBM Business Analytics, Tokyo, Japan). *p* values < 0.05 were considered statistically significant.

## 3. Results

Table 1 summarizes the characteristics of the subjects at the study entry year. The mean age was 45.1 years, geometric mean of HsCRP was 0.05 mg/dL, and geometric mean of AST was 21.1 IU/L.

Table 2 indicates the number of person-years and incidence rate. The number of subjects who developed liver damage was 800, and the incidence per 1000 person-years was 34.5. 

The odds ratios (OR) and 95% confidence intervals (CI) for the development of liver damage were shown in Table 3. The OR of a 1.5-fold increase in HsCRP levels for the development of liver damage was 1.07 (95% confidence interval: 1.03–1.10, *p* < 0.001). Thus, increased serum HsCRP levels was significantly associated with the development of liver damage. 

Alcohol consumption (OR(95% CI): 1.46 (1.35–1.59)), BMI (OR(95% CI): 1.10 (1.07–1.13)), mean blood pressure for each 10 mmHg increase (OR(95%CI): 1.16 (1.06–1.26)), and meals cooked by oneself (OR(95% CI): 1.39 (1.07–1.80)) had positive associations with the development of liver damage, while age (+1 y) (OR(95% CI):0.99 (0.98–0.99)) and a 1.5-fold increase in HDL-C (OR(95% CI): 0.87 (0.76–0.99)) had negative associations.

## 4. Discussion

The main finding was that an increase in HsCRP values was significantly associated with the development of liver damage in men after adjusting for various confounders. This is the first cohort study to show a causal association between increased HsCRP levels and the development of liver damage defined as elevated aminotransferase. This result is similar to previous research that reported the risk of developing NAFLD as determined by ultrasonography increased as the HsCRP level increased on the baseline among healthy middle-aged male workers in Korea [25]. However, liver ultrasonography is not routinely performed in healthy persons because it is usually undertaken to investigate abnormal liver function tests and costs more than routine liver function tests. Consequently, our finding has wider applications in primary health care.

Several cross-sectional studies have already documented an association between increased HsCRP levels and the development of liver damage. Recent studies partly supported our findings, but previous studies reported inconsistent result as follows. Elevated AST and increased CRP were associated independent of sex, age, components of MetS, and lifestyle-related factors in nonoverweight middle-aged Japanese men [17] and in middle-aged women with and without MetS [18]. However, another cross-sectional study noted a negative association between CRP and AST in middle-aged men with and without MetS [18], as well as in a case-control study in overweight and obese patients in a Belgian cohort [33]. Positive associations between alanine aminotransferase (ALT) and CRP have been reported in various populations, such as healthy subjects without any cardiovascular disease history [14], young healthy Japanese men without overweight [20], middle-aged male and female subjects in Israel [21], and men in the West Scotland [23]. Dose-response relationships were observed between gamma-glutamyltransferase and CRP in both nonoverweight and overweight middle-aged Japanese men [17] and in the third National Health and Nutrition Examination Survey [22]. Other studies have reported a positive association between alkaline phosphatase and CRP [19,21,33].

However, all but one of the above-noted studies were cross-sectional or case-control ones, therefore none of them could clarify any causal relationship. We have demonstrated, in a 6-year longitudinal study, an association between an increase in HsCRP levels and the development of liver damage a causal association can be considered between them.

Additionally, this study includes a greater number of subjects (7830 men) than earlier studies on inflammation and liver function [14,17,18,19,20,21,23,25] except for one cross-sectional study that clarified a positive association of serum gamma-glutamyltransferase and CRP in 12,110 adults in the U.S. [22].

The use of a pooled logistic regression analysis in this study is also one of its strengths. This analysis has an advantage compared with usual multivariable logistic regression analysis or Cox proportional hazards model analysis, in which only baseline HsCRP and covariates at baseline are considered. The annual health examinations were carried out from 2005 to 2010, and all of the updated data from this 6-year period could be used and evaluated by this analytical method. A pooled regression analysis is appropriate and very effective for analyzing data from multiple years and can adjust for covariates as well. We evaluate during each 1-year follow-up whether liver damage, defined as AST ≥ 40 IU/L, develops or not and consider any blood chemical test changes from the beginning of each 1-year follow-up to the end of the follow-up. Furthermore, CRP levels were used as a continuous variable in this study, resulting in no loss of information.

Subsequently, as we used a forward stepwise selection method, we could obtain an optimized statistical model consisting a minimum number of essential covariates. This information would be efficient and useful for similar evaluations in other populations as well. Furthermore, other studies have used fewer variables as potential factors than our study.

Many studies use explanatory variables, such as age, BMI, uric acid, various components of MetS, such as hypertension, dyslipidemia, hyperglycemia, waist circumference, presence of MetS per se, number of MetS features, and lifestyle-related factors, such as alcohol consumption and smoking. In addition, we investigated dietary habits, including meals (cooked by whom), soft drink consumption, and in-between snacking. Only one previous study adjusted for energy intake per day [17]. To our knowledge, job-related factors, such as job schedule and job stress have not previously been adjusted for before the present study.

In this study, elevated aminotransferase may be due to various etiologies of liver damage, including alcoholic liver disease (ALD), NAFLD, viral hepatitis, and rare liver diseases. In the U.S., the prevalence of aminotransferase elevation in men in the third National Health and Nutrition Examination Survey (1988–1994) [34] and the National Health and Nutrition Examination Survey (1999–2002) [35], respectively, were 9.3% and 9.3%. Likely etiologies of elevated aminotransferase levels were, in men and women, alcohol use (13.5%), hepatitis C (7.0%), possible hemochromatosis (3.4%), hepatitis B (0.9%), or a combination of causes (6.1%) [34]. In the setting of unknown etiology of elevated aminotransferase, 90% of cases had a histological diagnosis of fatty metamorphosis of the liver [36]. A Japanese group also reported that among subjects receiving health check-ups, liver dysfunction defined as ALT ≥ 40 IU/L was found in 15.4% in men. The most common cause of elevated ALT was fatty liver, with this accounting for liver dysfunction in 13.0% of males [37]. The reason for the causal association between increased HsCRP levels and liver damage defined as AST ≥40 IU/L is unclear. However, there are several possible explanations with speculation focusing especially on NAFLD.

Cellular stress induced by high-fat diet results in an increased production of IL-6, a proinflammatory cytokine, in adipocytes and macrophages and the concentration of circulating IL-6. In the liver, IL-6 inhibits the action of insulin, thereby promoting insulin resistance [38]. Indeed, in patients with NAFLD, hepatic IL-6 expression closely correlates with the degree of systemic insulin resistance, suggesting a possible mechanistic role for saturated free fatty acids in the increased hepatic IL-6 expression in NAFLD [39]. Another study reported that the intrahepatic gene expression of CRP was significantly elevated in nonalcoholic steatohepatitis patients as compared with simple steatosis patients, whereas IL-6 gene expression was not, suggesting an association between increased CRP and the severity of fatty liver [15]. It is well known that CRP is mainly expressed by the liver. In addition to that, it is found recently that it seems to also be produced in adipose tissue. Adipocytes are the source of a substantial portion of baseline IL-6 production and perhaps also synthesize and secrete some of the baseline CRP itself [12]. Because of the large amount of body fat present in obese patients, adipose tissue might contribute to increasing the serum CRP level [40]. Chronic low-grade inflammation, as manifested by especially the proinflammatory cytokines including IL-6 and TNF-α, might promote hepatic lipid accumulation through increased fatty acid uptake, enhanced TG synthesis, and reduced fatty acid oxidation in liver [41]. Hepatic fat infiltration in NAFLD may be influenced by visceral fat accumulation regardless of BMI [42]. In addition, Ndumele et al. documented an additive and independent association of hepatic steatosis with HsCRP levels, suggesting a connection between hepatic steatosis and systemic inflammation determining the risk of cardiovascular diseases [14]. Furthermore, the inflammatory microenvironment caused by steatosis leads to both amplification of steatosis and potentially the increased synthesis and release of CRP by the liver [24]. In patients with NAFLD and nonalcoholic steatohepatitis, adipose tissue insulin resistance and liver triglyceride content are major factors implicated in the elevation of plasma aminotransferase levels [43]. Taken all together, increasing serum CRP level may promote the development of liver damage via insulin resistance and interference with lipid metabolism.

In addition to increased CRP levels, BMI, alcohol consumption, mean blood pressure, and cooking meals by oneself compared with meals cooked by family showed positive associations with the development of liver damage, whereas HDL-C and age had negative associations. Our results were consistent with those of earlier studies, in which high BMI values [44,45] and excessive alcohol drinking [46,47] were positively associated with liver damage. Although it is difficult to make simple comparisons, the standard deviation of the alcohol consumption was 0.85, amounting to less than 1 gou (approximately 22 g of ethanol) in the participants of the present study, while the standard deviation of CRP was 2.9-fold, which is higher than the 1.5-fold increase in HsCRP. Therefore, the OR per standard deviation for alcohol consumption is considered to be greater, therefore the effect of alcohol consumption is assumed to be greater on the increase in AST than the 1.5-fold increase in CRP.

Hypertension is also related to liver damage, and in cross-sectional studies, NAFLD may be seen early in the development of hypertension, even in the absence of other metabolic risk factors [5]. The reason that cooking meals by oneself is associated with more frequent development of liver damage than cooking meals by family is unknown but may reflect a higher daily caloric intake or less well-balanced diet in the former. The reason why some factors showed higher ORs than the 1.5-fold increase in levels of HsCRP is not clear, but as shown in previous studies, each of them is a well-established factor in the development of liver dysfunction or a factor associated with elevated aminotransferases. This study showed the same trend as the previous studies as mentioned above.

Increased HDL-C levels had negative associations with the development of liver damage, suggesting that an increase in HDL-C may protect the liver [45]. The negative association of older age with the development of liver damage is in agreement with both a cross-sectional study [37] and a longitudinal study [48]. 

On the other hand, one limitation of this study was the absence of HBV and HCV serology. We did not have any information as to whether the subjects had or did not have a history of hepatitis B and C. However, in Japan, the estimated prevalence of subjects with HCV and HBV are 0.63% and 0.71%, respectively, in Japan. Furthermore, subjects in their 60s and older have a higher HCV prevalence, while subjects younger than about 50 have a lower HCV prevalence [49]. Thus, the impact of this factor is considered to be limited in our study population made up of middle-aged male subjects.

Furthermore, the main site of CRP production is the liver [12], therefore it is possible that another confounder, like other cytokines or pathways in the liver or adipocytes, may affect both the chronological increase of CRP and development of liver damage. To confirm any causal association between increased HsCRP levels and liver damage, further monitoring of this group or other cohorts should be conducted.

In addition, ALT is considered to be more specific to indicate liver injury than AST, which is also elevated in some diseases affecting other organs, such as heart and muscle [50]. Nevertheless, AST itself is also a useful indicator of liver damage in general [28,50]. Future studies should include analysis of ALT and longitudinal studies that combine AST and ALT as an outcome to more accurately assess liver damage.

Moreover, in this study, we focused on the new onset of liver injury as an outcome and conducted a longitudinal study. To further clarify the association, it would be of interest to see whether the level of HsCRP is positively associated with aminotransferase levels in patients with liver damage. Our research group has conducted longitudinal studies on HbA1c, BMI, and other parameters as related new onset of diabetes and obesity, as well as an outcome amounting to an increase of some percent as a continuous variable. We will consider a similar evaluation of the relationship between liver damage and HsCRP in the future.

## 5. Conclusions

The present study identified elevated HsCRP levels as an independent predictor of the development of liver damage in male Japanese workers using pooled logistic regression analysis after adjusting for confounders.

## Figures and Tables

**Table 1:** Characteristics of subjects at study entry year.

Variables	Mean (Standard Deviation)
Age (y)	45.1 (12.0)
Body mass index (kg/m^2^)	23.8 (3.2)
Systolic blood pressure (mmHg)	129.8 (13.1)
Diastolic blood pressure (mmHg)	78.9 (8.9)
Mean blood pressure (mmHg)	95.9 (9.6)
Alcohol consumption (gou */day)	0.75 (0.85)
	Geometric mean (Geometric standard deviation)
C-reactive protein (mg/dL)	0.05 (2.9)
Aspartate aminotransferase (IU/L)	21.1 (1.3)
High-density lipoprotein cholesterol (mg/dL)	52.9 (1.3)
HbA1c (%)	5.3 (1.1)
Creatinine (mg/dL)	0.8 (1.2)
Uric acid (mg/dL)	5.7 (1.3)
	Rate (%)
Job Schedule type	
Daytime	61.1%
3-shiftwork	32.1%
2-shiftwork	5.2%
Others	1.6%
Smoking	
Nonsmoker	46.3%
Smoker	53.7%
Habitual exercise	
None	37.6%
once-twice/month	18.9%
once-twice/week	26.9%
3–4 times/week	10.4%
5 times/week or more	6.2%
Meals	
Cooked by oneself	6.0%
Cooked by family	81.0%
Eating out or catering	6.0%
Eating in a dormitory	6.2%
Others	0.8%
Soft drink consumption	
Rare	28.0%
Occasional	46.3%
Frequent	25.6%
In-between snacking	
Rare	41.2%
Occasional	50.7%
Frequent	8.0%
Brief Job Stress Questionnaire	
Job demand (High)	43.7%
Job control (Low)	38.4%
Interpersonal relationship (Low)	17.6%
Compatibility (Low)	19.7%

* 1 gou (180 mL) contains approximately 22 g of ethanol.

**Table 2:** Number of person-years studied and incidence rate, grouped according to age at study entry.

Data Items	Men
Number of subjects examined	7830
Number of subjects who developed liver damage	800
(%)	10.2
Total person-years of observation	23,215
Incidence rate per 1000 person years	34.5
Mean observed years per person	2.96

**Table 3:** Odds ratios and 95% confidence intervals for the development of liver damage in men.

Men		
Independent Variables	OR * (95% CI ^†^)	*p*
C-reactive protein	1.07 (1.03, 1.10)	<0.001
Age (+1 y)	0.99 (0.98, 0.99)	<0.001
Alcohol consumption (+1 gou/day)	1.46 (1.35, 1.59)	<0.001
Body mass index (+1 kg/m^2^)	1.10 (1.07, 1.13)	<0.001
Mean blood pressure (+10 mmHg)	1.16 (1.06, 1.26)	0.001
High-density lipoprotein cholesterol	0.87 (0.76, 0.99)	0.034
Meals (/Cooked by family ^‡^)		
Cooking by oneself	1.39 (1.07, 1.80)	0.014
Eating out or catering	0.89 (0.65, 1.21)	0.459
Eating in a dormitory	0.86 (0.59, 1.26)	0.436
Others	1.68 (0.84, 3.36)	0.141

* Odds ratios adjusted for the effect of all other covariates using pooled logistic regression. ^†^ 95% confidence interval. ^‡^ Control categories. The data of C-reactive protein, high-density lipoprotein cholesterol, HbA1c, creatinine, and uric acid were logarithmically transformed using a base of 1.5. By forward stepwise method, selected potential covariates (which are shown in the above table) were adjusted for.

## Data Availability

The data that support the findings of this study are available from the corresponding authors upon reasonable request.
